# The Subtilisin-Like Protease AprV2 Is Required for Virulence and Uses a Novel Disulphide-Tethered Exosite to Bind Substrates

**DOI:** 10.1371/journal.ppat.1001210

**Published:** 2010-11-24

**Authors:** Ruth M. Kennan, Wilson Wong, Om P. Dhungyel, Xiaoyan Han, David Wong, Dane Parker, Carlos J. Rosado, Ruby H. P. Law, Sheena McGowan, Shane B. Reeve, Vita Levina, Glenn A. Powers, Robert N. Pike, Stephen P. Bottomley, A. Ian Smith, Ian Marsh, Richard J. Whittington, James C. Whisstock, Corrine J. Porter, Julian I. Rood

**Affiliations:** 1 Australian Research Council Centre of Excellence in Structural and Functional Microbial Genomics, Monash University, Clayton, Victoria, Australia; 2 Department of Microbiology, Monash University, Clayton, Victoria, Australia; 3 Department of Biochemistry and Molecular Biology, Monash University, Clayton, Victoria, Australia; 4 Faculty of Veterinary Science, University of Sydney, Camden, New South Wales, Australia; 5 Elizabeth Macarthur Agricultural Institute, NSW Department of Primary Industries, Camden, New South Wales, Australia; The Rockefeller University, United States of America

## Abstract

Many bacterial pathogens produce extracellular proteases that degrade the extracellular matrix of the host and therefore are involved in disease pathogenesis. *Dichelobacter nodosus* is the causative agent of ovine footrot, a highly contagious disease that is characterized by the separation of the hoof from the underlying tissue. *D. nodosus* secretes three subtilisin-like proteases whose analysis forms the basis of diagnostic tests that differentiate between virulent and benign strains and have been postulated to play a role in virulence. We have constructed protease mutants of *D. nodosus*; their analysis in a sheep virulence model revealed that one of these enzymes, AprV2, was required for virulence. These studies challenge the previous hypothesis that the elastase activity of AprV2 is important for disease progression, since *aprV2* mutants were virulent when complemented with *aprB2*, which encodes a variant that has impaired elastase activity. We have determined the crystal structures of both AprV2 and AprB2 and characterized the biological activity of these enzymes. These data reveal that an unusual extended disulphide-tethered loop functions as an exosite, mediating effective enzyme-substrate interactions. The disulphide bond and Tyr92, which was located at the exposed end of the loop, were functionally important. Bioinformatic analyses suggested that other pathogenic bacteria may have proteases that utilize a similar mechanism. In conclusion, we have used an integrated multidisciplinary combination of bacterial genetics, whole animal virulence trials in the original host, biochemical studies, and comprehensive analysis of crystal structures to provide the first definitive evidence that the extracellular secreted proteases produced by *D. nodosus* are required for virulence and to elucidate the molecular mechanism by which these proteases bind to their natural substrates. We postulate that this exosite mechanism may be used by proteases produced by other bacterial pathogens of both humans and animals.

## Author Summary

Extracellular proteases are produced by many bacterial pathogens and are commonly involved in the degradation of the host extracellular matrix, facilitating invasion and colonization. One such pathogen is *Dichelobacter nodosus*, the causative agent of ovine footrot, a disease of major economic significance to the international sheep industry. *D. nodosus* secretes a number of serine proteases, which are thought to cause the tissue damage associated with virulent footrot. Our study showed that a *D. nodosus* mutant lacking one of the three secreted proteases, AprV2, failed to cause virulent disease in sheep. We used x-ray crystallography to solve the structure of AprV2 and the closely related protease AprB2. Our structures revealed an unusual extended disulphide-tethered loop that is located next to, but does not form part of, the primary substrate binding site. Through targeted mutagenesis studies we were able to show that this loop functions as an exosite, mediating effective enzyme-substrate interactions. Bioinformatic analyses suggest that subtilases from other pathogenic bacteria may contain this loop and may therefore utilize a similar mechanism. Our multidisciplinary research approach has provided a comprehensive understanding of the functional role of extracellular proteases in the pathogenesis of ovine footrot.

## Introduction

*Dichelobacter nodosus* is a Gram negative, anaerobic rod that is the principal causative agent of ovine footrot, a debilitating disease of the hoof of ruminants. The disease results in significant costs to the worldwide sheep industry due to a reduction in meat and wool production and the expenditure associated with prevention and treatment programs [1], [2], [3]. Footrot is characterized by the separation of the keratinous hoof from the underlying tissue, resulting in severe lameness and loss of body condition [4], [5]. The severity of the disease can vary from benign footrot, which presents as an interdigital dermatitis that does not progress, to virulent footrot, which results in severe under-running of the horn of the hoof and the separation of the hoof from the underlying tissue [1]. Type IV fimbriae are an essential virulence factor [6], [7]; it also has been suggested that three closely-related secreted subtilisin-like proteases produced by *D. nodosus* may be required for virulence [8], [9]. In strains that cause virulent footrot these proteases are called acidic protease isoenzymes 2 and 5 from virulent strains (AprV2 and AprV5) and basic protease from virulent strains (BprV). In benign strains the comparable proteases are termed AprB2, AprB5 and BprB. All of these proteases are synthesised as inactive precursors with an N-terminal pre-pro-region, a serine protease domain and a C-terminal domain of unknown function. The active protease is produced by cleavage of the N-terminal pre-pro region and the C-terminal domain [10], [11], [12]. The protease domains have significant sequence identity to members of the subtilase family of serine proteases (54% identity with closest homologue from *Dehalococcoides sp. VS*), but sequence alignments indicate several insertions in the *D. nodosus* proteases [13].

Previous studies suggested that these proteases may represent the key difference between virulent and benign strains of *D. nodosus*; proteases secreted by virulent isolates have a greater thermostability and elastase activity (as monitored on elastin agar plates) than those of benign strains and it is postulated that this difference may relate to their *in vivo* activity against host tissue. These features are utilized in diagnostic tests to distinguish between virulent and benign footrot [14], [15], [16]. Comparison of the protease sequences from the virulent strain A198 with the benign strain C305 revealed that within the mature protease domain there is a single amino acid difference between AprV2 and AprB2 [12], and between AprV5 and AprB5 [11], and 96% sequence identity between BprV and BprB [17].

In this multidisciplinary study we set out to determine the role of these proteases in virulence and to determine the molecular basis for their function. We constructed isogenic protease mutants and characterized their protease activity and virulence. We showed that AprV2 was required for a virulent *D. nodosus* isolate to cause disease. Determination of the crystal structure of AprV2 revealed the presence of a novel exosite loop. This combined genetic and structural approach has permitted a comprehensive investigation of the secreted protease component of a pathogenic organism, and furthermore provided novel insight into how subtilisin-like proteases may have been hijacked by pathogenic microorganisms to degrade extracellular matrix components.

## Results

### Protease activity of *D. nodosus*

**Figure 1:**
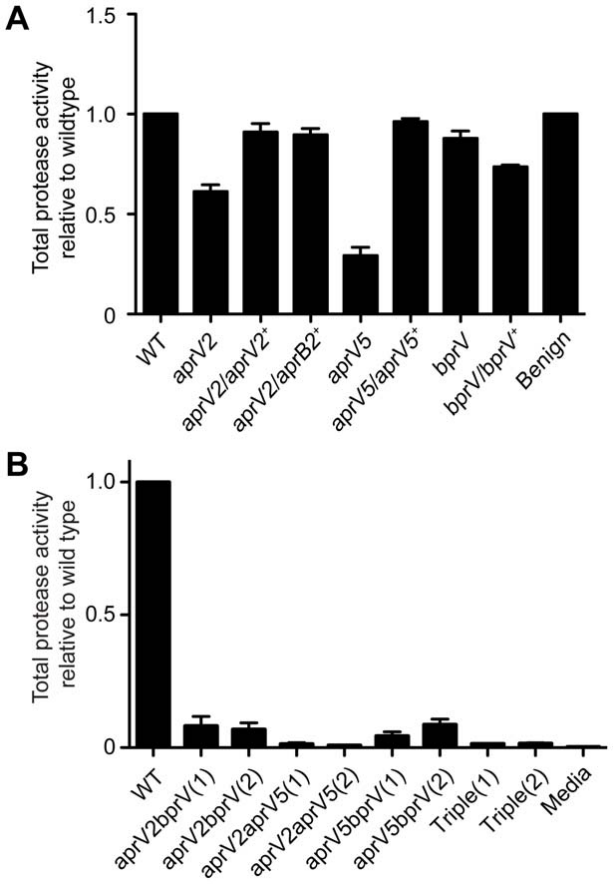
Protease activity of D. nodosus wild-type and protease mutants. Total protease activity of 40 h culture supernatants was measured using azocasein as the substrate. The protease activity is expressed relative to the wild-type activity. Means and standard error of the mean (s.e.m.) are shown. (**A**) Total protease activity of the protease mutants and their complemented derivatives. Each of the mutants had significantly reduced (*P*<0.05, n = 3, t-test) protease activity compared to the wild-type strain VCS1703A, while the complemented strains, except for the *bprV* complemented strain, were not significantly different to wild-type. WT: Wild-type VCS1703A, Benign: the benign isolate CS101. (**B**) Total protease activity of double and triple protease mutants. WT: Wild-type VCS1703A, Triple: the *aprV2 aprV5 bprV* triple mutant. The designation (1) and (2) represents independently derived mutants. Other genotypes are as indicated. The protease activity of each of these mutants was significantly different to wild-type (*P<*0.05, n = 3, t-test).

To assess the contribution of each of the three extracellular proteases to the overall protease activity of the virulent *D. nodosus* isolate VCS1703A, separate chromosomal mutants of each protease gene were constructed by allelic exchange events that involved double crossovers. To confirm that the observed phenotypes resulted from these mutations, the mutants were complemented by insertion of the wild-type protease genes into the chromosome. Quantitative protease assays of culture supernatants, using azocasein as the substrate, showed that mutation of the *aprV5* and *aprV2* genes reduced total protease activity by 71**%** and 39**%**, respectively (Figure 1A). Complementation with the respective wild-type genes returned total protease activity to wild-type levels; however, it was also observed that the complemented *aprV5* strain tended to lose protease activity upon repeated subculture. Since only a 12% reduction (*P<*0.05) was observed in the *bprV* mutant, BprV does not appear to make a major contribution to total protease activity. These results indicate that AprV5, either directly or indirectly, makes the major contribution to total extracellular protease activity.

To confirm the individual contribution of each protease gene to the overall protease activity of VCS1703A, double and triple mutants were also constructed. Only very low levels of protease activity were observed in the *aprV2bprV* and *aprV5bprV* double mutants (Figure 1B). Negligible protease activity was detected in the triple mutant and the *aprV2aprV5* double mutant. The reduction in total protease activity for the double mutants was greater than the combined reduction in the total protease activity for the single mutants suggesting that the secreted proteases either act synergistically to degrade target substrates or that one or more of the proteases may be involved in the activation of the other proteases, or both.

The ability of *D. nodosus* to digest insoluble elastin in an agar medium has been used as a diagnostic test to distinguish virulent and benign strains. Virulent isolates digest elastin within seven to ten days, while benign strains show no digestion after >21 days incubation [14]. Analysis of the wild type, the protease mutants and the complemented strains on elastin agar showed that the *aprV2* mutant was unable to digest elastin, even after 30 days incubation, whereas both the *aprV5* and *bprV* mutants were able to digest elastin at wild-type levels, showing clearing after 10 days (Figure S1A). Complementation of the *aprV2* mutation restored the ability to digest elastin. In addition, the *aprV5bprV* mutants still had elastase activity. These results provided evidence that the AprV2 protease was responsible for the elastase activity. Note that the levels of elastase activity in culture supernatants were not high enough for detection in quantitative elastase assays.

Sequence analysis of the *aprV2* and *aprB2* genes has shown there is only one amino acid difference (Y92R) between the two mature proteases [12]. To see what effect complementing the *aprV2* mutant with the *aprB2* gene would have on the protease phenotype of the resultant strain we inserted the *aprB2* gene from the benign strain CS101 into the site of the disrupted *aprV2* gene. Analysis of this strain in the azocasein assay showed that its total protease activity was not significantly different to the wild type (Figure 1A). However, this complemented strain had the *in vitro* phenotype of a benign strain since it had no elastase activity (Figure S1A), and its protease thermostability profile (Figure S1B) was that expected of a benign isolate [16].

**Figure 2:**
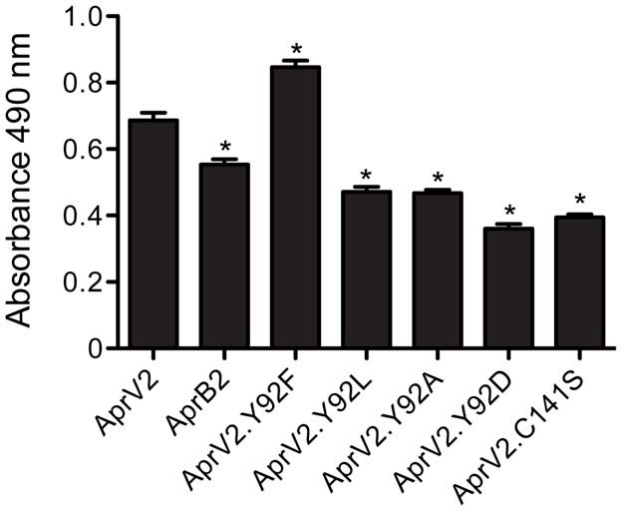
Elastase activity of AprV2, AprB2 and mutants. For quantitative measurement of elastase activity of recombinant AprV2, AprB2 (AprV2.Y92R) and protease mutants, purified protease was incubated with Elastin-Congo Red at 25°C for 19 h. Elastin degradation was detected spectroscopically at 490 nm. The mean and s.e.m are shown (* *p*<0.001, n = 3, one-way ANOVA compared to AprV2).

The apparent difference in elastase activity between AprV2 and AprB2 was assessed *in vitro* using an Elastin-Congo Red substrate and purified recombinant proteins. Under these conditions, the ability of AprB2 to degrade the substrate was significantly less than AprV2 (*p*<0.05, Figure 2). Both proteases displayed similar activity against the soluble chromogenic elastase substrate, N-Methoxysuccinyl-Ala-Ala-Pro-Val p-nitroanilide (AAPVn), with kinetic parameters that were of the same order of magnitude (Table 1). These results suggest that the Y92R substitution does not contribute directly to catalysis at the active site of the enzyme.

**Table 1:** Activity of AprV2, AprB2 and mutants against the elastin like peptide AAPV(n).

Proteases	V_max_ (µM.s^−1^)	*K*_M_ (mM)	k_cat_ (s^−1^)	k_cat_/*K*_M_ (M^−1^s^−1^)
AprV2	0.025±0.001	0.54±0.15	0.025±0.001	46.3
AprB2	0.033±0.001	0.41±0.08	0.033±0.001	80.5
AprV2.C141S	0.020±0.001	0.82±0.24	0.020±0.001	24.4
AprV2.Y92F	0.034±0.002	1.12±0.18	0.034±0.002	30.4
AprV2.Y92L	0.045±0.001	0.95±0.12	0.045±0.001	47.4
AprV2.Y92A	0.038±0.001	0.83±0.08	0.038±0.001	45.8
AprV2.Y92D	0.041±0.001	0.81±0.08	0.041±0.001	50.6

C141S is a substitution derivative of AprV2 in which one disulphide bond has been removed. AprB2 is equivalent to AprV2.Y92R. Experiments were conducted at 25°C using 1 µM purified protease.

### AprV2 is essential for virulence in sheep

**Figure 3:**
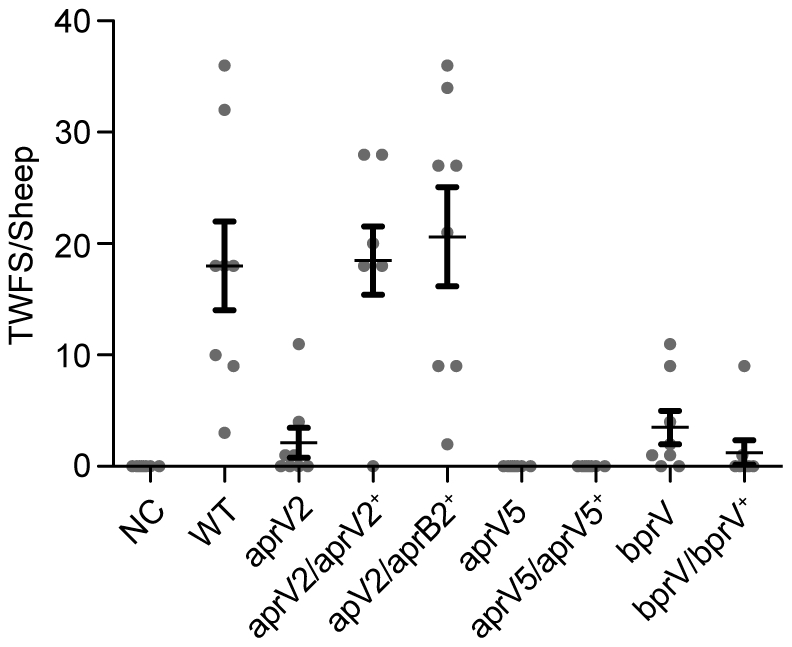
Virulence testing of isogenic isolates in sheep. Sheep were challenged with the wild type, the three protease mutants and their complemented derivatives in a blind pen trial. The total weighted foot score (TWFS) for individual sheep at week three is shown. The mean and the s.e.m. for each group are shown. Each mutant is significantly different to the wild-type, while the *aprV2/aprV2^+^* and *aprV2/aprB2^+^* complemented strains are not significantly different to wild-type (*P*<0.05, n = 8, one-way ANOVA). NC: negative control, not infected with bacteria.

To determine the role in disease of each of the proteases, virulence testing in sheep was carried out on the wild type, the *aprV2*, *aprV5* and *bprV* mutants and their corresponding complemented strains, using our standard procedure [6], [7]. These experiments represented a rigorous test of the ability of these bacteria to cause disease since such pen-based trials often magnify the ability of less virulent isolates to cause disease. Comparative analysis of the footrot scores of sheep infected with the wild-type strain and the *aprV2* mutant revealed a significant difference (*P*<0.0001); the *aprV2* mutant was effectively avirulent (Figure 3). Complementation of the *aprV2* mutant with the wild-type *aprV2* gene restored the wild-type virulence profile, fulfilling molecular Koch's postulates. To our surprise, complementation of the *aprV2* mutant with *aprB2* also restored a virulent phenotype, indicating that AprV2-mediated elastase activity was not required for virulence. Analysis of isolates obtained from infected lesions confirmed that they had the expected phenotypic and genotypic properties.

The *aprV5* and *bprV* mutants were also avirulent (Figure 3), but complementation of these strains with the respective wild-type genes did not restore virulence, which was unexpected. Extensive sequencing of each of the protease genes in these strains showed them to be intact. However, upon subculture, protease secretion and/or elastin digestion by the *aprV5* and *bprV* complemented strains were variable, as was their ability to undergo twitching motility, a property that is essential for virulence [7]. Therefore, we suggest that the complemented derivatives were genetically unstable and that secondary mutations were being selected in these strains. Consequently, no meaningful conclusions can be drawn from the *aprV5* and *bprV* sheep virulence experiments.

### Identification of the native substrates of AprV2

**Figure 4:**
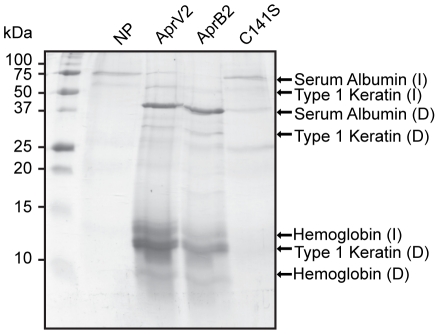
Degradation of sheep hoof by recombinant protease. The activity of AprV2, AprB2 and AprV2.C141S on fragments of hoof isolated from a disease free sheep. C141S is a substitution derivative of AprV2 in which one disulphide bond has been removed. The degradation products were visualised by SDS-PAGE. Degraded proteins were identified by in-gel tryptic digest followed by LCMS. I: intact proteins; D: degraded proteins; NP: no protease added.

Since the elastase activity of AprV2 was not required for virulence it was of interest to determine which hoof proteins were degraded by Aprv2 and AprB2. Fragments of hoof from a disease-free sheep were exposed to recombinant AprV2 and AprB2 and solubilised proteins were identified. AprV2 degraded type I keratin, serum albumin and the beta subunit of haemoglobin (Figure 4). Importantly, the hoof digestion pattern produced by AprB2 was similar to that produced by AprV2 (Figure 4), which is consistent with results of the virulence trials.

### AprV2 contains a novel disulfide tethered extended loop

**Figure 5:**
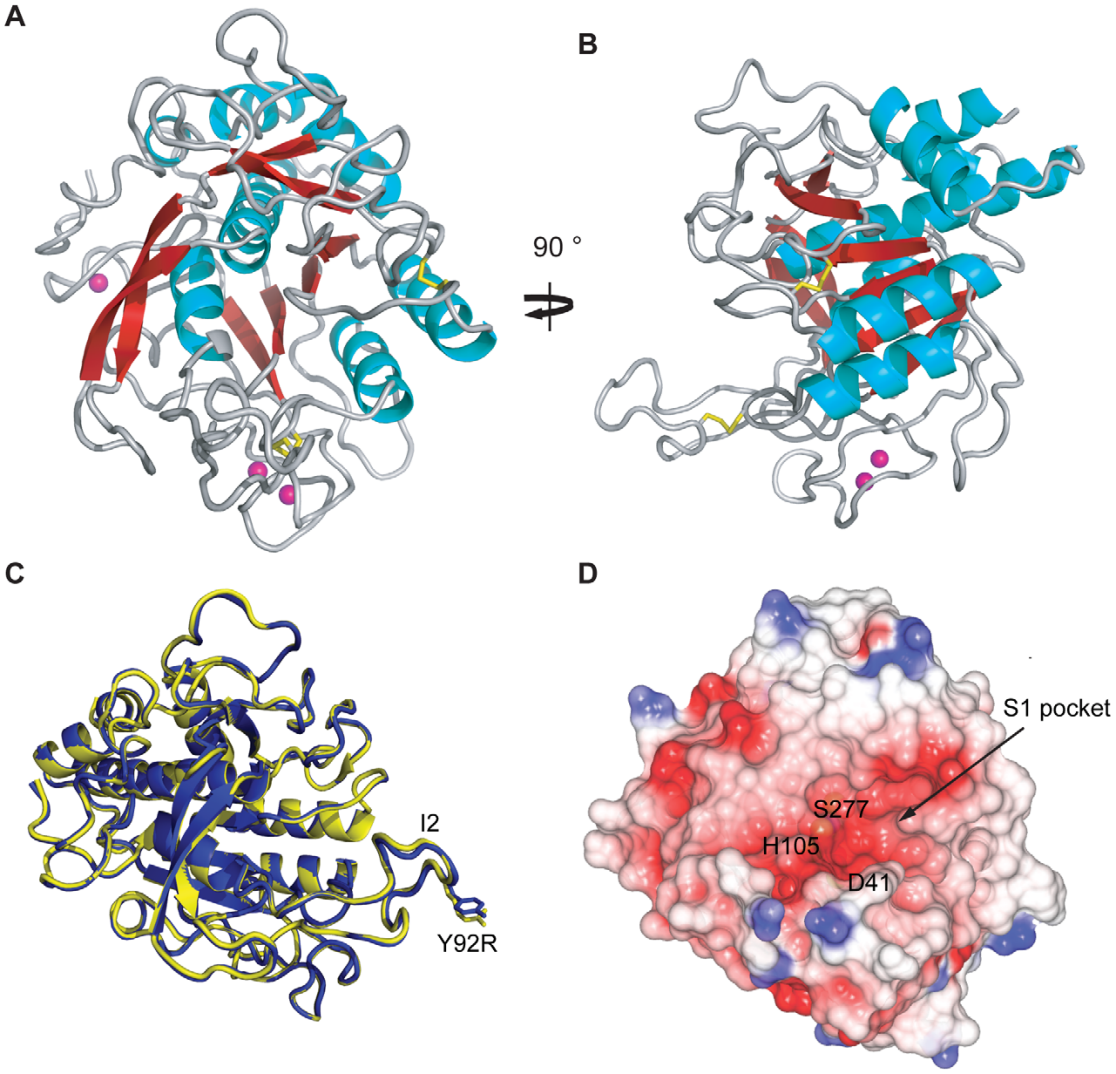
Crystal structure of AprV2. (**A**) and (**B**) Cartoon representation of AprV2. Two views differing by 90° are shown. Disulphide bonds are shown in yellow stick representation. Calcium ions found in the crystal structure are shown as pink spheres. (**C**) An overlay of the crystal structures of AprV2 (blue) and AprB2 (yellow). The structures are shown as a C_α_ trace. The I2 loop is labelled. Tyr 92 in AprV2 and Arg 92 in AprB2 are shown in stick representation and labelled. (**D**) Electrostatic potential surface of AprV2 generated using CCP4mg [50]. Positively charged electrostatic potential is coloured blue and negatively charged electrostatic potential is coloured red. The location of the active site residues is indicated. The location of the S1 binding pocket is indicated. (A), (B) and (C) were prepared using PyMol [51]. The structural alignment in (C) was prepared using MUSTANG [52]. Secondary structure elements were calculated using stride [53].

To investigate how the single amino acid difference (Y92R) between the active forms of AprV2 and AprB2 alters the substrate specificity of AprB2 we determined the crystal structures of AprV2 and AprB2 to 2.0 Å and 1.7 Å, respectively (Table 2; Figure 5A,B). The two structures were very similar (RMSD of 0.28 Å for 339 C_α_) (Figure 5C) and therefore we will describe the structure with reference to AprV2.

**Table 2:** Structure refinement statistics for AprV2, AprB2 and AprV2.C141S.

	AprV2	AprB2	AprV2.C141S
Resolution (Å)	24.3–2.0	20.4–1.7	24.1–2.1
No. reflections	16,749	25,598	14,360
*R*_work_/*R*_free_ a	17.7/23.3	13.8/17.8	18.2/23.7
No. atoms			
Protein	2495	2498	2468
Ligand/ion (Ca^2+^)	3	3	3
Water	155	371	81
*B*-factors			
Protein	14.9	12.4	17.2
Ligand/ion (Ca^2+^)	21.8	11.0	33.2
Water	17.8	26.3	17.9
r.m.s. deviations			
Bond lengths (Å)	0.012	0.011	0.011
Bond angles (°)	1.343	1.229	1.229
Ramachandran plot (%)			
Favoured region	96.2	96.7	95.3
Allowed region	3.8	3.3	4.4
Disallowed region	0	0	0.3
MOLPROBITY score	1.85	1.51	1.75

a R  =  ∑ |F_obs_ − F_cal_|/∑F_obs_, where R_free_ is calculated with the 5% of data omitted from the refinement and R_cryst_ with the remaining 95% of the data included in the refinement.

r.m.s.: root-mean-square.

AprV2 adopts a subtilisin-like fold consisting of a curved six-stranded parallel beta sheet sandwiched between two and five alpha helices (Figure 5A, B). A two stranded anti-parallel beta hairpin runs perpendicular to the plane of the central beta sheet. The proteases have two disulphide bonds, Cys89-Cys141 and Cys183-Cys220 and three calcium binding sites (Figure S2). The proposed catalytic triad (Asp41, His105 and Ser277) of AprV2 is located at the C-terminal edge of the beta sheet. The most striking feature of the substrate binding site is a large, elongated S1 binding pocket, which is lined by residues 177–180, 204–208, 215 and 218, and appears capable of accommodating bulky side-chains such as phenylalanine (Figure 5D). This finding is consistent with previous studies, which have shown that AprV2 preferentially cleaves after phenylalanine or leucine residues [18].

**Figure 6:**
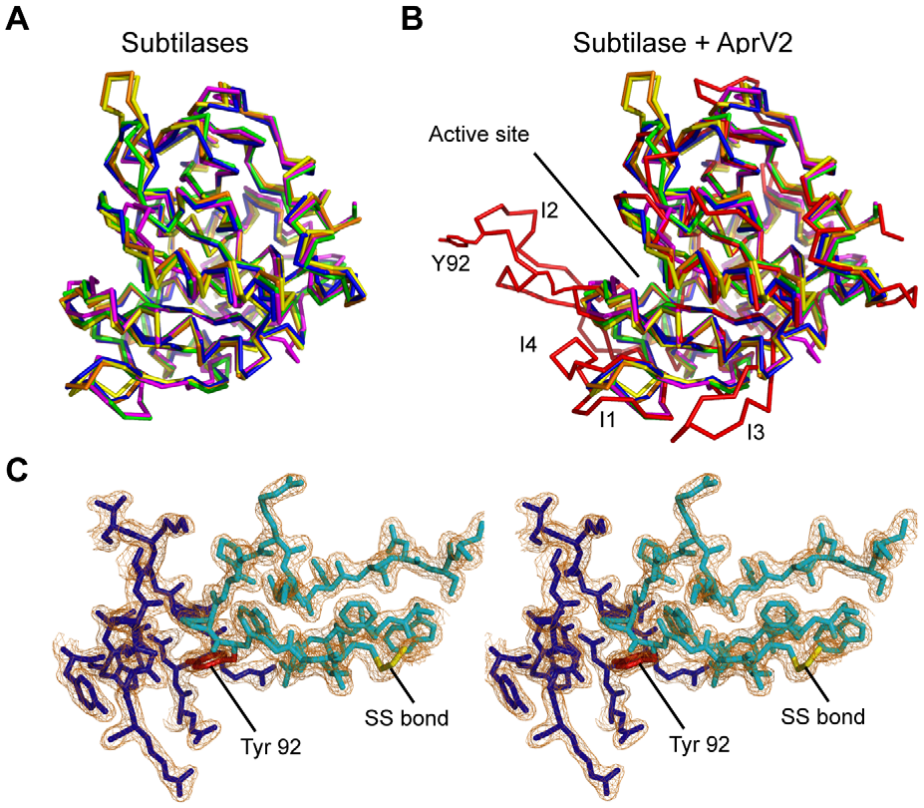
AprV2 contains a novel disulfide tethered loop. (**A**) Overlay of the crystal structures of AkP (magenta; 1DBI), thermitase (green; 1THM), Carlsburg subtilisn (yellow; 1AF4), BPN' (orange; 1SUP) and savinase (blue; 1SVN). (**B**) Overlay of the crystal structures of AprV2 (red) with AkP, thermitase, Carlsburg subtilisn, BPN' and savinase (coloured as in (a)). The structures were superimposed using the A chains only and are shown as a C_α_ trace. The I1, I2, I3 and I4 loops are labelled. Tyr 92 in AprV2 and Arg 92 are shown in stick representation and labelled. (**C**) Stereo view of a 2|F_o_|−|F_c_| electron density map depicting the disulfide tethered I2 loop of the AprV2 protease. The map is contoured at 1.2 σ. Water molecules have been removed for clarity. The conformation of this loop (cyan) is stabilised by inter and intramolecular contacts with molecules generated by symmetry coloured blue. The alignments in (**A**) and (**B**) were generated using MUSTANG [52]. Secondary structure elements were calculated using stride [53]. The figure was prepared using PyMol [51].

Comparison with other subtilisin-like proteases reveals several major insertions (termed I1-I4) in the loops that surround the active site cleft (Figure 6A, B; Figure S3). Most notable is the large well ordered I2 loop (residues 82–102) that is tethered to the subtilisin-like fold by a disulphide bond between Cys141 and Cys89 (Figure 6). The loop is well defined in the electron density, with low B-factors, suggesting that it has limited mobility in the crystal structure (Figure 6C). However, the apparent stability of the I2 loop is likely to arise from crystal packing and it is uncertain if this conformation would be favoured in solution. Surprisingly, the single amino acid difference between AprV2 and AprB2 (Y92R) is located at the tip of this extended loop, ∼27 Å from the active site serine (S277). A PSI-BLAST search revealed that the additional loops present in AprV2 may be conserved in other extracellular proteases (Figure S3B).

We constructed an I2 loop truncation mutant, AprV2Δ_83–99_, but the resultant protein was not functional, therefore we investigated the role of the I2 loop using site-directed mutagenesis. We targeted residue 92 as the Y92R substitution reduced the ability of AprB2 to degrade Elastin-Congo Red (Figure 2), while maintaining its ability to degrade the elastin-like peptide AAPVn (Table 1). We used site-directed mutagenesis to convert Tyr92 to Asp, Ala, Leu or Phe and examined the ability of the resultant proteins to degrade insoluble Elastin-Congo Red. While the presence of a negative charge (Asp), positive charge (Arg) or smaller hydrophobic (Ala and Leu) side-chain decreased elastin degradation, the Phe substitution increased the elastase activity of the enzyme (Figure 2). We also examined the ability of these mutants to degrade AAPVn (Table 1), fibronectin (Figure S4) or hoof material (Figure S5). No major differences were discernable. Based on these data we conclude that an aromatic ring is required at position 92 for maximal activity against insoluble elastin, but that this activity is not related to the hoof digestion observed in a footrot lesion.

### The Cys89-Cys141 disulphide bond is required for optimal activity

**Figure 7:**
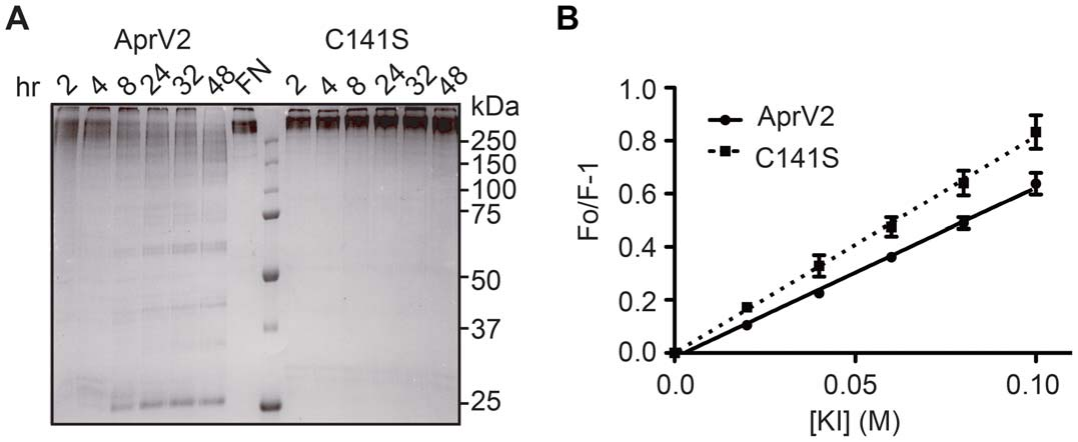
Characterisation of AprV2.C141S derivative. (**A**) Degradation of fibronectin by purified AprV2 and AprV2.C141S at 25°C. The degradation of fibronectin over time was assessed by SDS-PAGE analysis. (**B**) Stern-Volmer plot for iodide quenching of tryptophan in AprV2 and AprV2.C141S. Purified protease was incubated with increasing amounts of KI, and the fluorescence emission intensity was recorded. The lines represent a least squares fit of the experimental data as described previously [49]. The mean and s.e.m from three independent experiments are shown. Note that in these figures AprV2.C141S is shown as C141S.

To test the importance of the Cys89-Cys141 disulfide bond for proteolytic activity, we also used site-directed mutagenesis to convert Cys141 to Ser141. Although this AprV2.C141S derivative was still active against small peptide substrates (Table 1), we noted that the ability of this enzyme to degrade fibronectin, insoluble elastin and proteins from sheep hoof was reduced. Notably, wild-type AprV2 was able to break down fibronectin in 48 h, whereas at the corresponding time point AprV2.C141S-treated fibronectin was still intact (Figure 7A). The elastinolytic activity of the C141S protease was also approximately two-fold lower than wild type (Figure 2). Finally, the ability of AprV2.C141S to degrade sheep hoof material was significantly reduced compared to AprV2 and AprB2 (Figure 4). Together these data suggest that the integrity of the I2 loop and the Cys89-Cys141 disulfide bond is important for maximal AprV2 protease activity.

**Figure 8:**
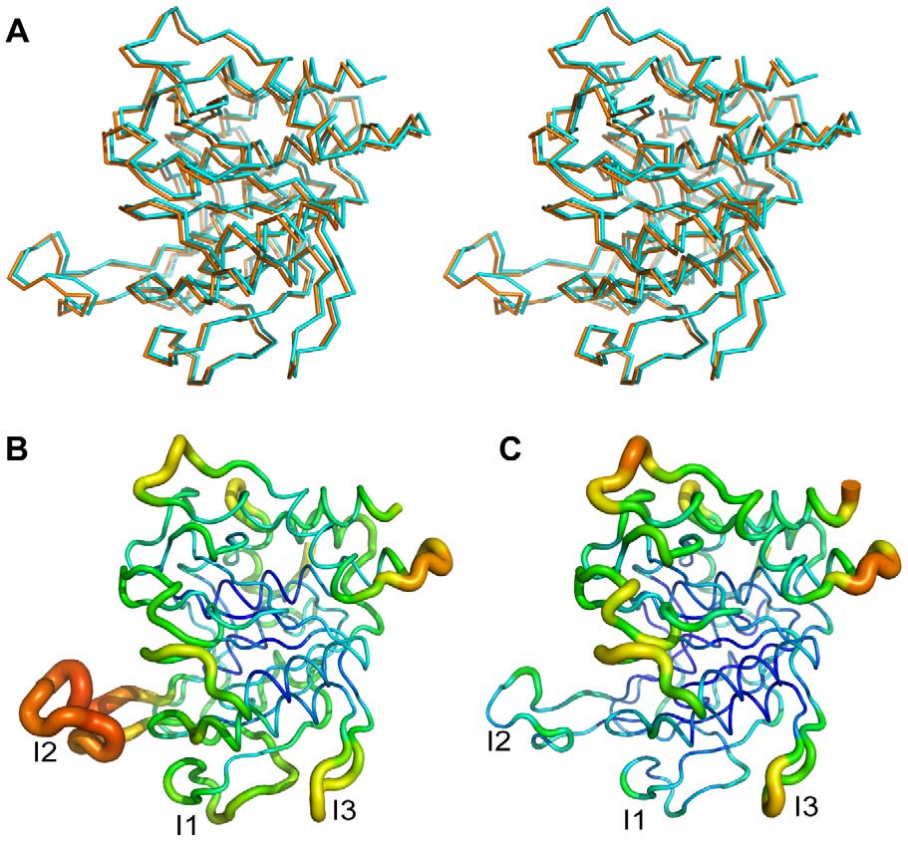
Crystal structure of AprV2.C141S. (**A**) Overlay of AprV2 (orange) with AprV2.C141S (cyan) shown in stereo. The structures are shown as a C_α_ trace. (**B**) and (**C**) Ribbon representation of AprV2.C141S (**B**) and AprV2 (**C**) coloured and sized according to B-factor. High B-factors are coloured red and shown as thicker ribbon. Low B-factors are coloured blue and shown as thinner ribbon. The alignment was generated using the program MUSTANG [52]. The figure was prepared using Pymol [51].

To determine whether the C141S substitution affects the conformation/mobility of the I2 loop we determined the crystal structure of AprV2.C141S to 2.1 Å (structure refinement statistics in Table 2). The structure of AprV2.C141S was very similar to that of AprV2, overlaying with an RMSD of 0.21 Å for 339 C_α_. The structure confirmed the absence of the Cys89-Cys141 disulfide bond and revealed that while the I2 loop had a slightly different structure to that of AprV2 there were no significant differences in the structure of the active site or primary substrate binding site (Figure 8A and S6). The average B factors for the I2 loops in AprV2 and AprV2.C141S were 14.6 Å^2^ and 28.4 Å^2^, respectively, indicating that the I2 loop is more mobile in the substituted protease (Figure 8B,C). Given that the conformation of the I2 loop is stabilized by crystal packing in all three structures we investigated whether the I2 loop was more mobile in solution in AprV2.C141S using intrinsic tryptophan fluorescence spectroscopy; the I2 loop contains two tryptophans. Steady state fluorescence quenching data showed that the wild-type protease was more protected from quenching by potassium iodide than the C141S enzyme (Figure 7B), confirming that the conformation of the I2 loop in AprV2.C141S is different to that in the wild type.

## Discussion

Although the extracellular proteases of *D. nodosus* have been considered for many years to be potential virulence factors [9], their importance in the pathogenesis of disease had not been established. The analysis of their role in disease has always been complicated by the fact each isolate produces three very closely related proteases. The genetic approach utilized here has now provided clear evidence that the AprV2 protease is essential for virulence. This conclusion is based on data that showed that an *aprV2* mutant was unable to cause footrot in sheep, unlike the isogenic wild-type strain, and that the ability to cause disease was restored to wild-type levels in the complemented derivative. No definitive conclusions could be drawn from the virulence testing of the *aprV5* and *bprV* mutants. However, it is likely that the AprV5 and BprV proteases also play a role in disease, especially since the three secreted proteases appear to act synergistically, with the double protease mutants, *aprV2aprV5*, *aprV2bprV*, and *aprV5bprV*, showing lower secreted protease activity than that expected based on the secreted protease activity of the individual mutants (Figure 1B). It remains to be elucidated how this synergism arises, although it could occur at the processing, secretion or substrate level.

Benign and virulent strains of *D. nodosus* can be differentiated by phenotypic analysis of their extracellular proteases, including analysis of their elastase activity and thermostability [14], [16], [19], [20]. We now have established that AprV2 is responsible for the elastase activity of the virulent isolate VCS1703A. Purified AprB2, which differs in sequence from AprV2 only at residue 92, is less efficient at degrading elastin. Therefore, it was important to examine the effect on virulence of complementing the *aprV2* mutant with *aprB2*. This AprV2^−^AprB2^+^ strain was benign by the standard laboratory tests used to differentiate virulent and benign strains. Unexpectedly, it was virulent in the sheep footrot trial, producing disease that was indistinguishable in footrot severity from that caused by the isogenic wild-type strain. Since this strain still produces both AprV5 and BprV we conclude that the presence of one benign protease (AprB2) in combination with two virulent proteases (AprV5 and BprV) is not sufficient to make a strain benign even though in a laboratory diagnostic context the strain would be designated as benign. Therefore, although it appears that the properties of AprV2 and AprB2 are responsible for the differentiation of benign and virulent strains in laboratory tests, there clearly are other virulence factors, such as the other virulent proteases, that contribute to virulent disease.

We have shown that AprV2 mediates the degradation of keratin (Figure 4), a component of the ovine hoof that confers physical protection and tissue integrity. This finding suggests that AprV2 has a direct role in destroying the keratin layer of the ovine hoof, a characteristic feature of virulent footrot. The initial site of *D. nodosus* attachment during infection is at the epidermal layer of the interdigital skin and degradation of keratin in this area by AprV2 is likely to be required to break through the skin horn junction, allowing the subsequent under-running of the horn by *D. nodosus*.

An intriguing feature identified in the structures of AprV2 and AprB2 is the disulphide-tethered I2 loop. This loop is located next to, and partially occludes, the substrate binding site of the enzyme (Figure 6). The single amino acid difference between AprV2 and AprB2 is located at the tip of the loop. We have shown that residue 92 is a key determinant of elastase activity. Although, substitution of Tyr92 with Arg reduced the degradation of insoluble elastin, no significant differences in either *K*_M_ or V_max_ were observed for hydrolysis of the elastin-like peptide, AAPVn. This result suggests that residue 92 does not contribute to catalysis at the active site of the enzyme. Instead, the reduced elastinolytic activity of AprB2 is likely to arise from impaired enzyme-substrate interactions at a site distal to the active site. We therefore propose that the I2 loop functions as an exosite, mediating the formation of a stable enzyme-substrate complex. The disulphide bond tethering the I2 loop appears to be important for this function since its disruption alters the mobility of the loop, which significantly reduces the ability of the protease to degrade fibronectin, insoluble elastin and other proteins from the sheep hoof.

We have identified several serine proteases that like AprV2 also appear to contain large insertions between the β1 strand and α2 helix (the location of the I1 and I2 loops) (Figure S3). These enzymes include MprA, from *Burkholderia pseudomallei* (the causative agent of melioidosis), TgSUB1 and TgSUB2, from *Toxoplasma gondii* (the causative agent of toxoplasmosis) and PfSUB1 from the malaria parasite *Plasmodium falciparum*. MprA degrades physiologically relevant proteins and may play a role in causing the lung damage associated with melioidosis [21], [22], however, it has only a minor role in virulence [23]. PfSUB1 and TgSUB2 appear to be critical for parasite survival [24], [25]. The presence of the I2-like insertions in these proteins suggests that the mechanism of exosite loop-mediated proteolysis used by the *D. nodosus* secreted proteases may represent a mechanism of substrate recognition that is utilized by other bacterial proteases. Studies investigating the structure of these proteins along with the function of their I2-like insertions will shed some light on this hypothesis and may lead to the development of improved diagnostic reagents and the identification of novel vaccine and drug targets.

## Materials and Methods

### Construction and characterization of *D. nodosus* mutants and complemented strains

Strains and plasmids are detailed in Table 3. *D. nodosus* strains were routinely grown in an anaerobic chamber (Coy Laboratory Products Inc.) as described previously [6].

**Table 3:** Bacterial strains and plasmids.

Plasmids	Genetic Characteristics	Properties	Source or Reference
**Strains**			
***E. coli***			
DH5α	F^−^ *endA1 hsdR17*(r_k_^−^m_k_)*thi-1λ^−^recA1 gyrA96relA1 rhoA supE44 deoRФ80dlacZ*ΔM15*Δ(lacZYA argF)*U169		Invitrogen
RosettaGami(DE3). pLysS	*Δ(ara-leu)7697 ΔlacX74 ΔphoA PvuII phoR araD139 ahpC galE galK rpsL* (DE3) F'*[lac+ lacIq pro] gor522::Tn10 trxB* pLysSRARE (CamR, StrR, TetR)		Novagen
***D. nodosus***		**Protease produced**	
VCS1703A	*D. nodosus* serogroup G wild-type, virulent	AprV2, AprV5, BprV	J. Egerton, University of Sydney
CS101	*D. nodosus* serogroup G wild-type, benign	AprB2, AprB5, BprB	D. Stewart, CSIRO Livestock Industries
JIR3740*, JIR3743*	VCS1703A*aprV2*Ω*tet*(M)	AprV5, BprV	Double crossover from pJIR2097
JIR3756*, JIR3757*	VCS1703A*aprV5*Ω*bla*	AprV2, BprV	Double crossover from pJIR2309
JIR3766*, JIR3767*	JIR3740*bprV*Ω*erm*(B)	AprV5	Double crossover from pJIR2275
JIR3768*, JIR3769*	JIR3757*aprV2*Ω*bla*	BprV	Double crossover from pJIR2097
JIR3883	JIR3756*rrnA*Ω*aprV5^+^*	AprV2, AprV5, BprV	Double crossover from pJIR2545
JIR3900	JIR3743Δ*erm*(B)Ω*aprV2^+^tet*(M)	AprV2, AprV5, BprV	Double crossover from pJIR3044
JIR3903*, JIR3905*	JIR3743Δ*aprV5bprVΩerm(B)*	None	Double crossover from pJIR3060
JIR3907*, JIR3908*	VCS1703AΔ*aprV5bprVΩerm(B)*	AprV2	Double crossover from pJIR3060
JIR3923	JIR3743Δ*erm*(B)Ω*aprB2^+^tet*(M)	AprB2, AprV5, BprV	Double crossover from pJIR3268
JIR3928	VCS1703A*bprV*Ω*erm*(B)	AprV2, AprV5	Double crossover from pJIR3159
JIR3930	JIR3928Δ*erm*(B)Ω*bprV^+^*Kan^R^	AprV2, AprV5, BprV	Double crossover from pJIR3148
**Plasmids**		**Properties**	
pUC18	Ap^r^ *lacZ* cloning vector		[54]
pWSK29	Ap^r^ *lacZ* low copy number cloning vector		[55]
pWKS30	Ap^r^ *lacZ* low copy number cloning vector		[55]
pWSK129	Km^r^ *lacZ* low copy number cloning vector		[55]
pET22b	Ap^r^ *lacI* cloning vector harbouring T7 promoter and terminator with pBR322 and f1 origin.		Novagen
pJIR2097	pWSK29 harbouring 5.2 kb fragment containing *aprV2 Ω tetM*	*aprV2* suicide plasmid	Recombinant
pJIR2275	pUC18 harbouring 3.4 kb fragment containing *bprV Ω erm* (B)	*bprV* suicide plasmid	Recombinant
pJIR2309	pWSK129 harbouring 3.5 kb fragment containing *aprV5Ω bla*	*aprV5*suicide plasmid	Recombinant
pJIR2545	pWKS30 harbouring 4.8 kb fragment containing *D. nodosus rrnA* promoter, kanamycin resistance, *aprV5, rrnA* terminator	*aprV5* complementation plasmid	Recombinant
pJIR3044	pWSK29 harbouring 5.9 kb fragment containing *aprV2, erm*(B) and 2.3 kb chromosomal region downstream of *aprV2*	*aprV2* complementation plasmid	Recombinant
pJIR3060	pWSK29 harbouring 3.75 kb fragment containing 5′fragment of *aprV5, erm*(B) and 3′fragment of *bprV*	*aprV5/bprV* suicide plasmid	Recombinant
pJIR3148	pWSK29 harbouring 4.3 kb fragment containing 3′ fragment of *aprV5*, kanamycin resistance, *bprV*	*bprV* complementation plasmid	Recombinant
pJIR3159	pWSK29 harbouring 3.5 kb fragment containing *bprV Ω erm*(B)	*bprV* suicide plasmid	Recombinant
pJIR3268	pWSK29 harbouring a 5.9 kb fragment containing *aprB2, erm*(B) and 2.3 kb chromosomal region downstream of *aprV2*	*aprB2* complementation plasmid	Recombinant
pET22b-AprV2	pET22b harbouring 1.4 kb fragment encoding 1–474 residues of the AprV2 precursor.	AprV2 expression	Recombinant
pET22b-AprB2	pET22b harbouring 1.4 kb fragment encoding 1–474 residues of the AprB2 precursor.	AprB2 expression	Recombinant
pET22b-AprV2.Y92F	pET22b harbouring 1.4 kb fragment encoding 1–474 residues of the AprV2 precursor with a Y92F substitution.	AprV2.Y92F expression	Recombinant
pET22b-AprV2.Y92D	pET22b harbouring 1.4 kb fragment encoding 1–474 residues of the AprV2 precursor with a Y92D substitution.	AprV2.Y92D expression	Recombinant
pET22b-AprV2.Y92L	pET22b harbouring 1.4 kb fragment encoding 1–474 residues of the AprV2 precursor with a Y92L substitution.	AprV2.Y92L expression	Recombinant
pET22b-AprV2.Y92A	pET22b harbouring 1.4 kb fragment encoding 1–474 residues of the AprV2 precursor with a Y92A substitution.	AprV2.Y92A expression	Recombinant
pET22b-AprV2.C141S	pET22b harbouring 1.4 kb fragment encoding 1–474 residues of the AprV2 precursor with a C141S substitution.	AprV2.C141S expression	Recombinant

*Independently derived mutants.

To construct the single mutants, suicide plasmids were inserted into *D. nodosus* strain VCS1703A by natural transformation [6]. The *aprV2* and *bprV* mutants were complemented by transforming the mutants with the relevant plasmids, which reconstituted the disrupted gene and inserted a different resistance marker. The *aprV5* mutant was complemented by inserting an intact copy of *aprV5* and a kanamycin resistance marker into one of the three *rrnA* operons. The *aprV2bprV* double mutant was constructed by inserting the *bprV* suicide plasmid into an *aprV2* mutant, and the *aprV2aprV5* double mutant constructed by inserting the *aprV2* suicide plasmid into an *aprV5* mutant. An *aprV5bprV* double mutant was constructed by inserting a suicide plasmid into the wild-type strain, which disrupted both genes when a double crossover event occurred. Finally, a triple *aprV2aprV5bprV* mutant was constructed by inserting the *aprV5bprV* suicide plasmid into the *aprV2* mutant.

All mutants and complemented strains were confirmed by PCR and Southern hybridizations. PCR-RFLP analysis of the *omp* gene family was used to confirm that mutants were derived from the wild-type strain [26]. Elastase activity and protease thermostability assays for the differentiation of benign and virulent strains of *D. nodosus* were as described previously [14], [16], [27].

### Virulence testing in sheep

Virulence testing in sheep was performed as before [6], [7]. The sheep were randomly allocated into nine groups of eight sheep and challenged blind with the various strains. A plain agar challenge was used as the negative control. The feet of all animals were examined and scored for footrot lesions at the start of the trial and then at weekly intervals using a standard lesion scoring method [28], [29]. The total weighted foot score (TWFS) was used to provide an unambiguous overall footrot score for each animal [29]. The trial was carried out in a PC2 containment facility at Elizabeth Macarthur Agricultural Institute in accordance with the guidelines of the Australian Government Office of the Gene Technology Regulator and the Elizabeth Macarthur Agricultural Institute Animal Ethics Committee.

### Measurement of protease activity

All assays were performed in 20 mM Tris-HCl pH 8 and 5 mM CaCl_2_ (buffer A) with the exception of the Elastin-Congo Red elastase assay, which was performed in 25 mM Bis-Tris pH6.5, 150 mM NaCl, 5 mM CaCl_2_ and 5% glycerol. Quantitative determination of total protease activity or elastase activity was carried out using azocasein [26] or Elastin-Congo Red [30] assays, respectively. Degradation of AAPVn by recombinant protease (1 µM) was measured at 25°C as described [31]. *K*_M_ and V_max_ were determined by plotting initial velocities against AAPVn concentration and fitted by non-linear regression (Prism). Fibronectin degradation was measured by incubating human fibronectin (1 µM, BD Biosciences) with recombinant protease (0.1 µM) at 25°C. Cleavage products were visualised by SDS-PAGE. Proteolytic degradation of hoof was determined by incubating dissected hoof material (2.2% (w/v) in buffer A) from a disease-free sheep with recombinant protease (110 µg/ml) at 25°C. Samples were taken over a 16 hour period and degradation products were visualised by SDS-PAGE.

### Proteolytic digestion of hoof and substrate identification by in-gel tryptic digestion and LCMS

Hoof material (14% (w/v) in buffer A) from a disease-free sheep was incubated with 100 µg of recombinant protease at 25°C for 18 h. Degradation products were separated by SDS-PAGE. The bands were excised and subjected to in-gel tryptic digestion and the digests analysed by LC-MS/MS using a HCT ULTRA ion trap mass spectrometer (BrukerDaltonics) coupled online with a 1200 series capillary HPLC (Agilent technologies). Proteins were identified by searching the LC-MS/MS data against the National Center for Biotechnology Information (NCBI) non-redundant and Swiss-Prot databases using the MASCOT search engine (version 2.1, Matrix Science Inc.) with all taxonomy selected.

### Protein production, crystallisation and data collection

AprV2 and AprB2 were purified and crystallised as before [32]. Data collection statistics have been reported [32]. The expression construct for AprV2.C141S was generated using the Quikchange site-directed mutagenesis kit (Stratagene) and pET22b.AprV2 as the template. Expression, purification and crystallisation of AprV2.C141S were as for AprV2. Data collection statistics for AprV2.C141S are in Table S1.

### Structure determination and refinement

Unless stated otherwise, all programs used for structural and crystallographic analysis were located within the CCP4 interface [33] to the CCP4 suite [34]. Manual building and maximum likelihood refinement were carried out using COOT [35] and REFMAC5 [36], respectively. The protease structures were solved by molecular replacement using PHASER [37]. A search model for AprB2 was derived from the coordinates of *Bacillus* Ak.1 protease (PDB code 1DBI [38]), identified using the FFAS server [39]. The search model was generated using the SCRWL server and consisted of all conserved side-chains with the remaining non-alanine/glycine residues truncated at the C^γ^ atom [40]. The initial model of AprB2 was subject to several iterations of manual building and refinement. The model was then subjected to automatic building using ARP/wARP [41] before the structure was completed by more cycles of manual building and refinement. The refined AprB2 structure with the I2 loop deleted was used as the MR search model for AprV2 and AprV2.C141S. The initial models were subjected to simulated annealing using PHENIX [42], [43]. Successive rounds of manual building and refinement incorporating TLS [44] generated the final models. Water molecules were added to all models using ARP/warp v 5.0 [45], [46]. Structure validation was carried out using MolProbity [47] and COOT [35]. Refinement statistics for the structures determined are presented in Table 2. The coordinates and structure factors are available from the Protein Data Bank (2LPA; 2LPC; 2LPC). Raw data and images are available from TARDIS (www.tardis.edu.au) [48].

### Fluorescence quenching experiments

Recombinant protease (0.5 µM in buffer A) was incubated with increasing amounts of quenching solution (2 M KI and 1 mM Na_2_S_2_O_3_) and the change in the fluorescence emission intensity of the tryptophan residues (λ_ex_290 nm/λ_em_340 nm) was measured using a Perkin-Elmer LS50B spectrofluorometer. The data were analysed as before [49].

### Ethics statement

The sheep virulence experiments were carried out in a PC2 containment facility at the Elizabeth Macarthur Agricultural Institute in accordance with the guidelines of the Australian Government Office of the Gene Technology Regulator and the Elizabeth Macarthur Agricultural Institute Animal Ethics Committee. These experiments were approved by the Elizabeth Macarthur Agricultural Institute Animal Ethics Committee.

## Supporting Information

Figure S1 Characterisation of the *D. nodosus* wild-type and protease mutants. **(A)** Elastase activity of protease mutants and the complemented strains. Elastase activity was determined by growing the strains indicated on TAS agar containing 0.3% elastin for 28 days. A zone of clearing of the insoluble elastin around the growth streak indicates elastase activity. **(B)** Protease thermostability of protease mutants and complemented strains. Culture supernatants were diluted 1:5 in Hepes Buffer (5.6 mM Hepes acid, 113.6 mM Hepes sodium salt, 10 mM CaCl_2_, 0.1 mM Zwittergent 3–14, pH 8.5 at 40°C) and 20 μl aliquots placed into wells in a gelatin-agarose gel after heating at 67°C for 0, 8 and 16 min [16]. Gels were then incubated at 37°C in a moist chamber overnight and then immersed in hot saturated ammonium sulphate to precipitate undigested protein. Zones of clearing around wells indicate protease activity.
(5.02 MB TIF)

Figure S2 Calcium binding sites in AprV2. Stereo diagrams showing the calcium binding sites in AprV2. Calcium ions are indicated as blue spheres, water molecules are indicated as red spheres. **(A)** The Ca-I binding site. This site corresponds to the high affinity A-site in the archetypal subtilisin BPN′ [56]. The Ca2^+^ ion is coordinated to the side chain oxygen atoms of Asp5 (O^δ1^), Asp49 (O^δ1^ and O^δ2^) and Asn119 (O^δ1^) and the carbonyl oxygen atoms from Val116, Ile121 and Val123. **(B)** The Ca-II binding site. The Ca2^+^ ion coordinates to four side chain oxygen atoms from Asp69 (O^δ1^ and O^δ2^), Asp71 (O^δ1^) and Asp74 (O^δ1^), two main chain carbonyl oxygen atoms from Asp71 and Gly72 and a water molecule. This site is unique to AprV2 and AprB2. **(C)** The Ca-III binding site. The Ca2^+^ ion is more solvent exposed than the other bound Ca ions and coordinates to the side chain oxygen atoms of Asp59 (O^δ2^), Asp69 (O^δ2^) and Asp74 (O^δ1^ and O^δ2^) along with the carbonyl oxygen atom of Asp76 and two water molecules. All calcium ions form a distorted pentagonal, bipyrimidal coordination geometry. The figure was prepared using PyMol [51].
(1.47 MB TIF)

Figure S3 The I2 loop. **(A)** AprV2 contains a novel disulfide tethered extended loop. Structure based sequence alignment of AkP (1DBI), thermitase (TM, 1THM), Carlsburg subtilisin (CBG, 1AF4), BPN′ (1SUP) and savinase (SAV, 1SVN) with AprV2 and AprB2. Secondary structure elements present in AprV2 are shown above the alignment. Residue numbering is for AprV2. The filled arrows indicate active site residues. The open arrow indicates the oxyanion hole. The I1, I2, I3 and I4 loops are labeled. Residues highlighted in red indicate strictly conserved residues. Residues in red text indicate those with similar physiochemical properties. The alignment was generated using the program MUSTANG [52] and ESPript [57]. Secondary structure elements were calculated using stride [53]. **(B)** Subtilisin-like proteases from pathogens that contain an I2-like insertion. Sequence alignment of AprV2 (*D. nodosus*), MprA (*B. pseudomallei*), PfSUB1 (*P. falciparum*), TgSUB1 (*T. gondii*), TgSUB2 (*T. gondii*), XC (*X. campestris*), PP (*P. piscicida*) and BPN′ (*B. amyloliquefaciens*). Secondary structure elements present in AprV2 are shown above the alignment. Residue numbering is for AprV2. Residues highlighted in red indicate strictly conserved residues. Residues written in red indicate those with similar physiochemical properties. The alignment was generated using the program Clustal W [58] and ESPript [57]. Secondary structure elements were calculated using stride [53].
(1.42 MB TIF)

Figure S4 Degradation of fibronectin by AprV2 and mutants. Purified protease was incubated with fibronectin at 25°C. The degradation of fibronectin over time was monitored by SDS-PAGE analysis. Intact fibronectin (FN) is >250 kDa. Degradation products are observed at molecular weight <250 kDa.
(5.02 MB TIF)

Figure S5 Degradation of sheep hoof by AprV2 and mutants. Purified protease was incubated with hoof material from a disease free sheep at 25°C. The degradation over time was monitored by SDS-PAGE analysis. Degradation products were observed between 5–15 kDa. RP: recombinant protease; HDP: hoof degradation products.
(5.95 MB TIF)

Figure S6 AprV2.C141S is structurally similar to AprV2. The crystal structure of AprV2.C141S and AprV2 were superimposed using LSQKAB in CCP4 [33], [34]. The C_α_ RMSD for each residue is plotted. The secondary structure of each residue is indicated with α-helices shown in dark grey and β-strands shown in black. The loop insertions are shown in grey and labelled. The location of the catalytic triad (Asp41, His105 and Ser277) and the disrupted disulphide bond (Cys89 and Cys141) is labelled. Secondary structure elements were calculated using stride [53].
(2.89 MB TIF)

Table S1 Data collection statistics for AprV2.C141S.
(0.05 MB DOC)
