# Supplementary figures and images for: The Subtilisin-Like Protease AprV2 Is Required for Virulence and Uses a Novel Disulphide-Tethered Exosite to Bind Substrates
Source: PLoS Pathog. 2010 Nov 24;6(11):e1001210. doi: 10.1371/journal.ppat.1001210 (PMC2991261; doi:10.1371/journal.ppat.1001210)

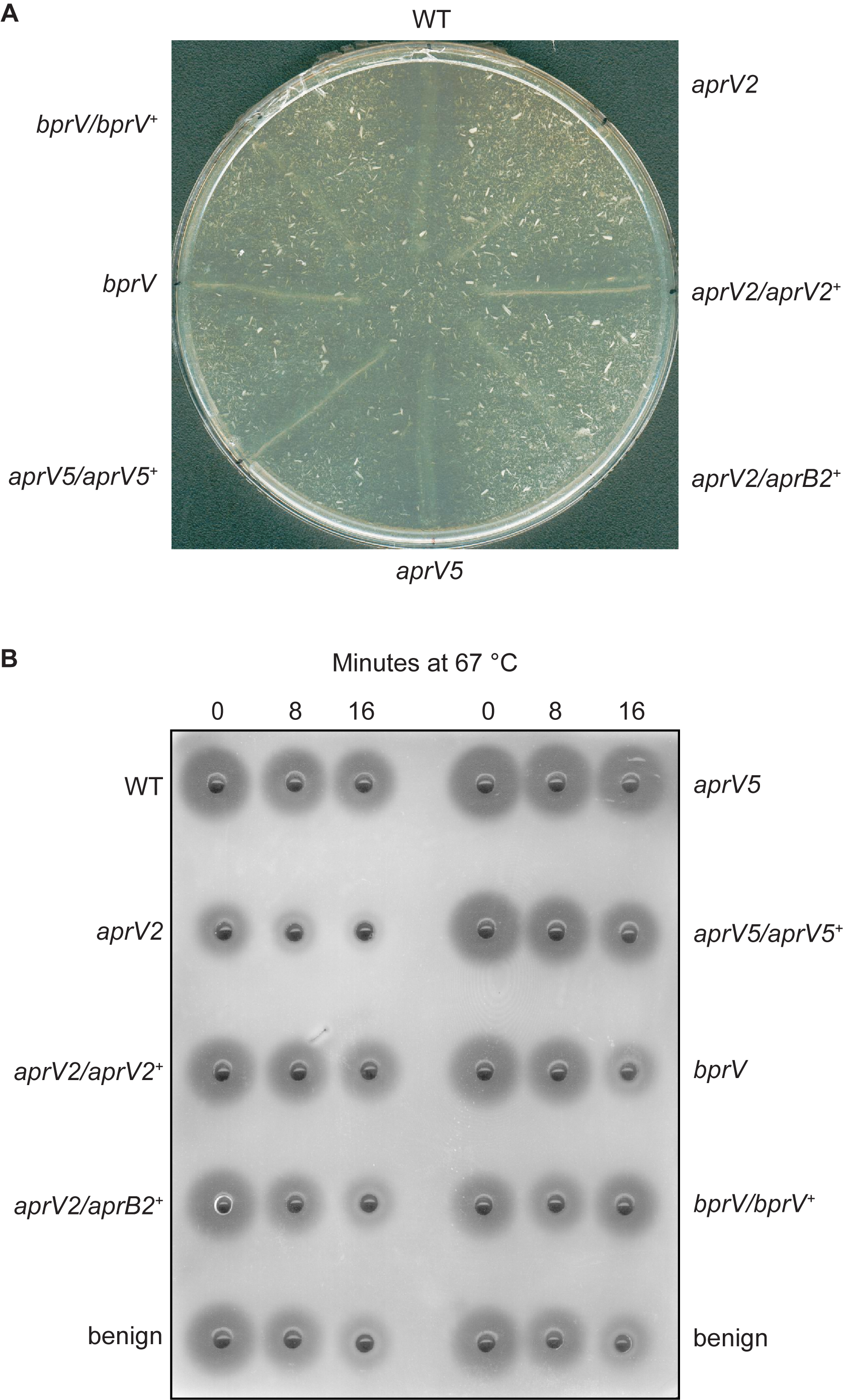

Supplement: Figure S1 — Characterisation of the D. nodosus wild-type and protease mutants. (A) Elastase activity of protease mutants and the complemented strains. Elastase activity was determined by growing the strains indicated on TAS agar containing 0.3% elastin for 28 days. A zone of clearing of the insoluble elastin around the growth streak indicates elastase activity. (B) Protease thermostability of protease mutants and complemented strains. Culture supernatants were diluted 1:5 in Hepes Buffer (5.6 mM Hepes acid, 113.6 mM Hepes sodium salt, 10 mM CaCl2, 0.1 mM Zwittergent 3–14, pH 8.5 at 40°C) and 20 μl aliquots placed into wells in a gelatin-agarose gel after heating at 67°C for 0, 8 and 16 min [16]. Gels were then incubated at 37°C in a moist chamber overnight and then immersed in hot saturated ammonium sulphate to precipitate undigested protein. Zones of clearing around wells indicate protease activity. (5.02 MB TIF) [file ppat.1001210.s001.tif]

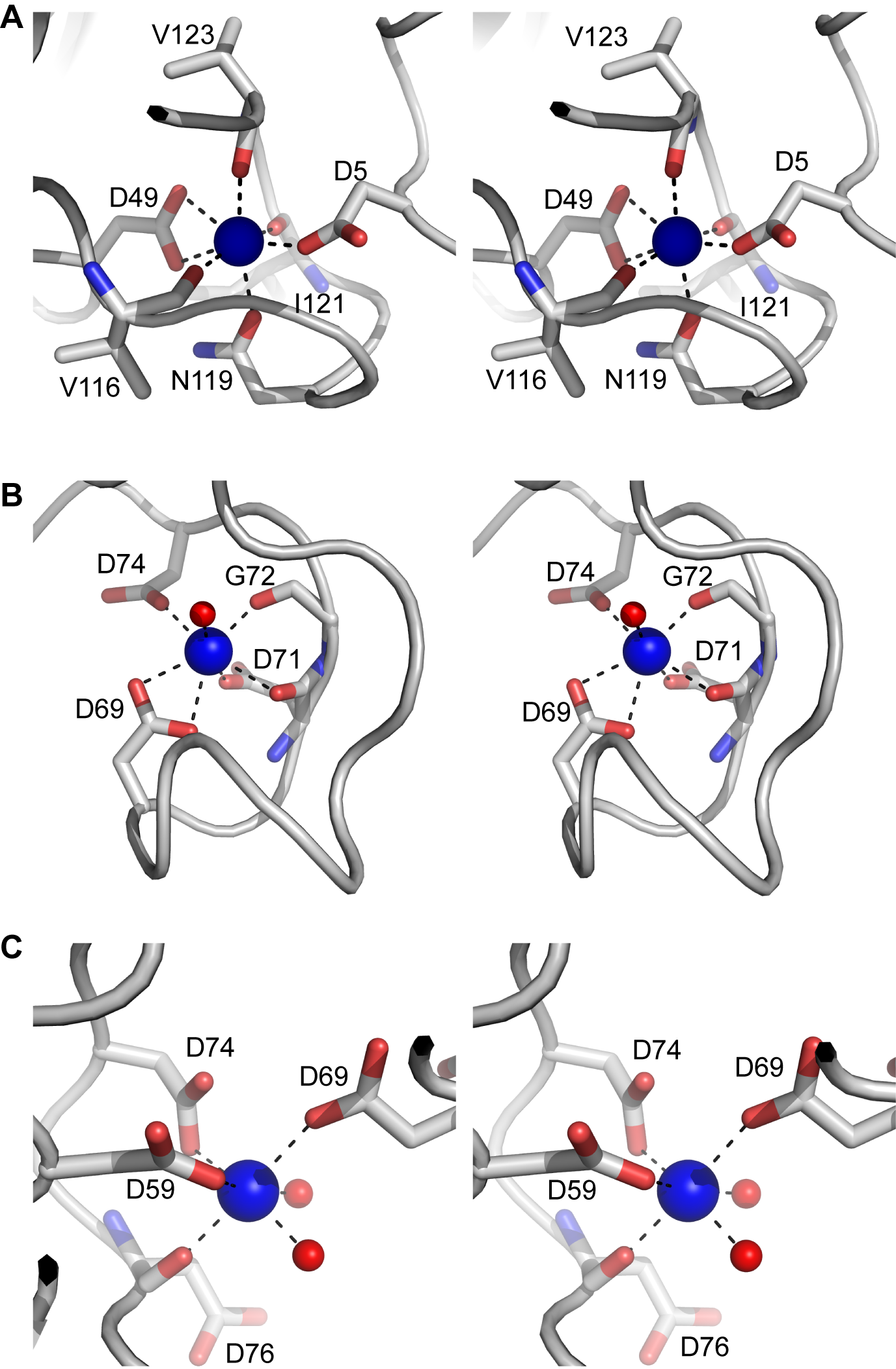

Supplement: Figure S2 — Calcium binding sites in AprV2. Stereo diagrams showing the calcium binding sites in AprV2. Calcium ions are indicated as blue spheres, water molecules are indicated as red spheres. (A) The Ca-I binding site. This site corresponds to the high affinity A-site in the archetypal subtilisin BPN′ [56]. The Ca2+ ion is coordinated to the side chain oxygen atoms of Asp5 (Oδ1), Asp49 (Oδ1 and Oδ2) and Asn119 (Oδ1) and the carbonyl oxygen atoms from Val116, Ile121 and Val123. (B) The Ca-II binding site. The Ca2+ ion coordinates to four side chain oxygen atoms from Asp69 (Oδ1 and Oδ2), Asp71 (Oδ1) and Asp74 (Oδ1), two main chain carbonyl oxygen atoms from Asp71 and Gly72 and a water molecule. This site is unique to AprV2 and AprB2. (C) The Ca-III binding site. The Ca2+ ion is more solvent exposed than the other bound Ca ions and coordinates to the side chain oxygen atoms of Asp59 (Oδ2), Asp69 (Oδ2) and Asp74 (Oδ1 and Oδ2) along with the carbonyl oxygen atom of Asp76 and two water molecules. All calcium ions form a distorted pentagonal, bipyrimidal coordination geometry. The figure was prepared using PyMol [51]. (1.47 MB TIF) [file ppat.1001210.s002.tif]

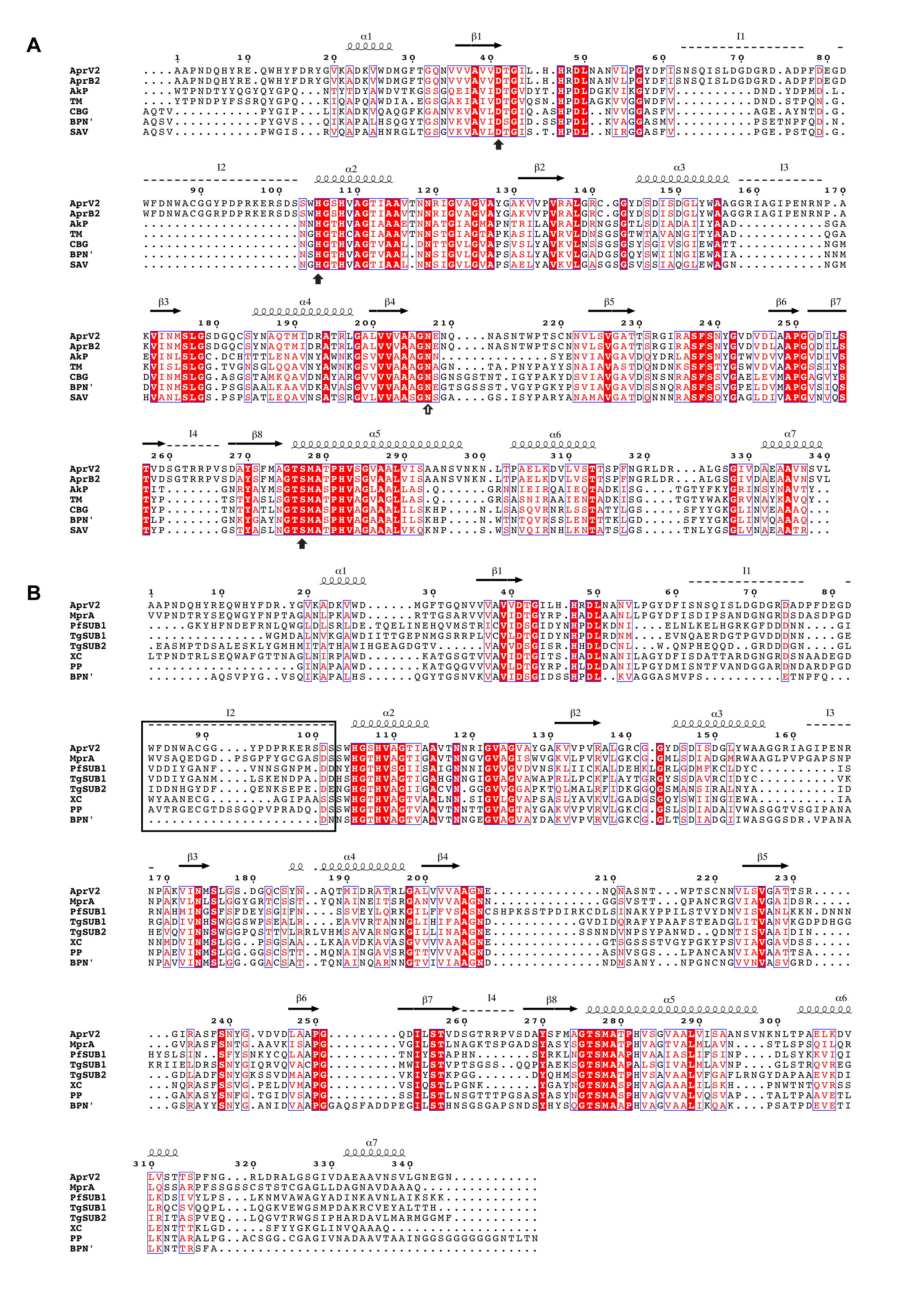

Supplement: Figure S3 — The I2 loop. (A) AprV2 contains a novel disulfide tethered extended loop. Structure based sequence alignment of AkP (1DBI), thermitase (TM, 1THM), Carlsburg subtilisin (CBG, 1AF4), BPN′ (1SUP) and savinase (SAV, 1SVN) with AprV2 and AprB2. Secondary structure elements present in AprV2 are shown above the alignment. Residue numbering is for AprV2. The filled arrows indicate active site residues. The open arrow indicates the oxyanion hole. The I1, I2, I3 and I4 loops are labeled. Residues highlighted in red indicate strictly conserved residues. Residues in red text indicate those with similar physiochemical properties. The alignment was generated using the program MUSTANG [52] and ESPript [57]. Secondary structure elements were calculated using stride [53]. (B) Subtilisin-like proteases from pathogens that contain an I2-like insertion. Sequence alignment of AprV2 (D. nodosus), MprA (B. pseudomallei), PfSUB1 (P. falciparum), TgSUB1 (T. gondii), TgSUB2 (T. gondii), XC (X. campestris), PP (P. piscicida) and BPN′ (B. amyloliquefaciens). Secondary structure elements present in AprV2 are shown above the alignment. Residue numbering is for AprV2. Residues highlighted in red indicate strictly conserved residues. Residues written in red indicate those with similar physiochemical properties. The alignment was generated using the program Clustal W [58] and ESPript [57]. Secondary structure elements were calculated using stride [53]. (1.42 MB TIF) [file ppat.1001210.s003.tif]

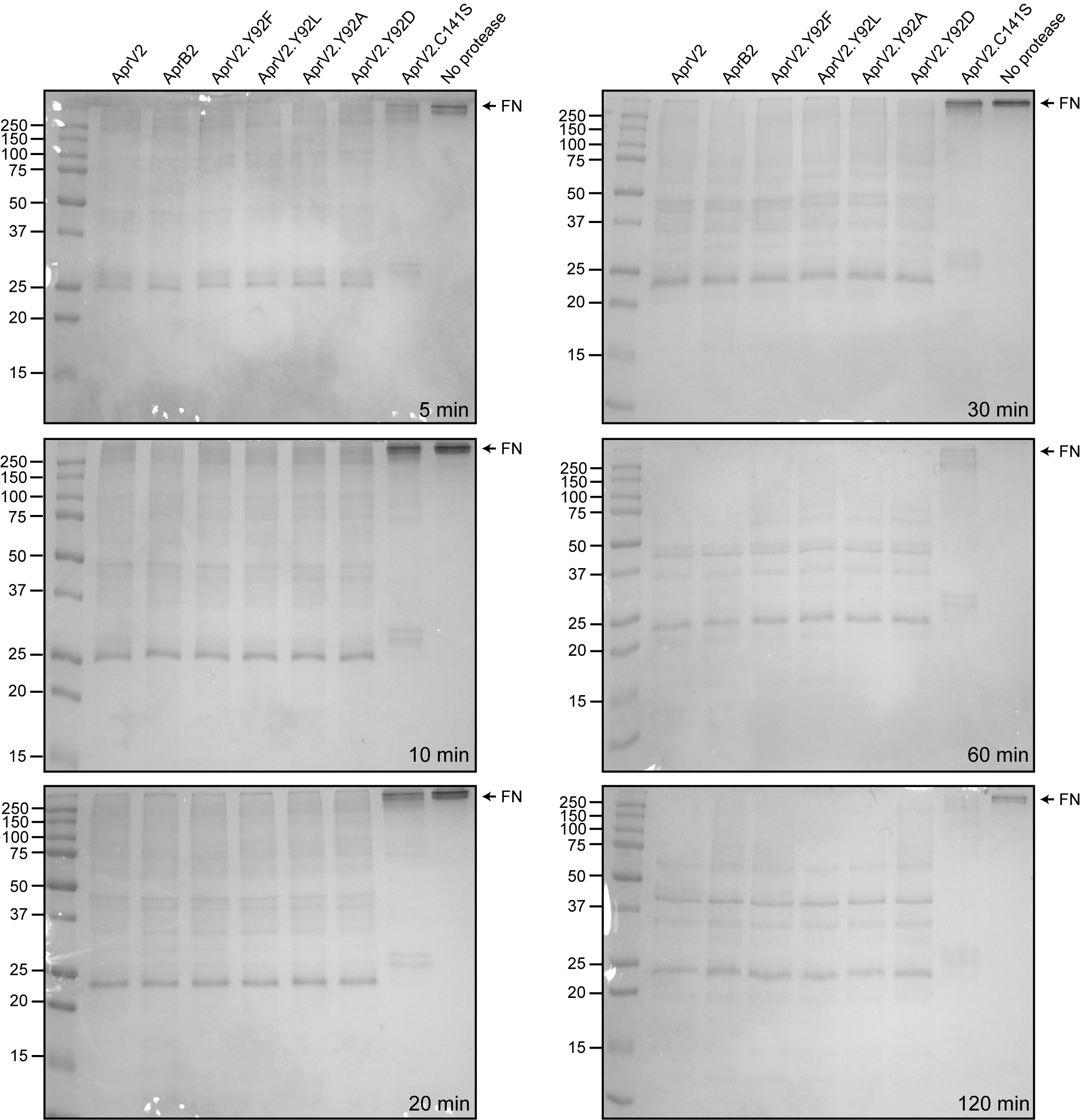

Supplement: Figure S4 — Degradation of fibronectin by AprV2 and mutants. Purified protease was incubated with fibronectin at 25°C. The degradation of fibronectin over time was monitored by SDS-PAGE analysis. Intact fibronectin (FN) is >250 kDa. Degradation products are observed at molecular weight <250 kDa. (5.02 MB TIF) [file ppat.1001210.s004.tif]

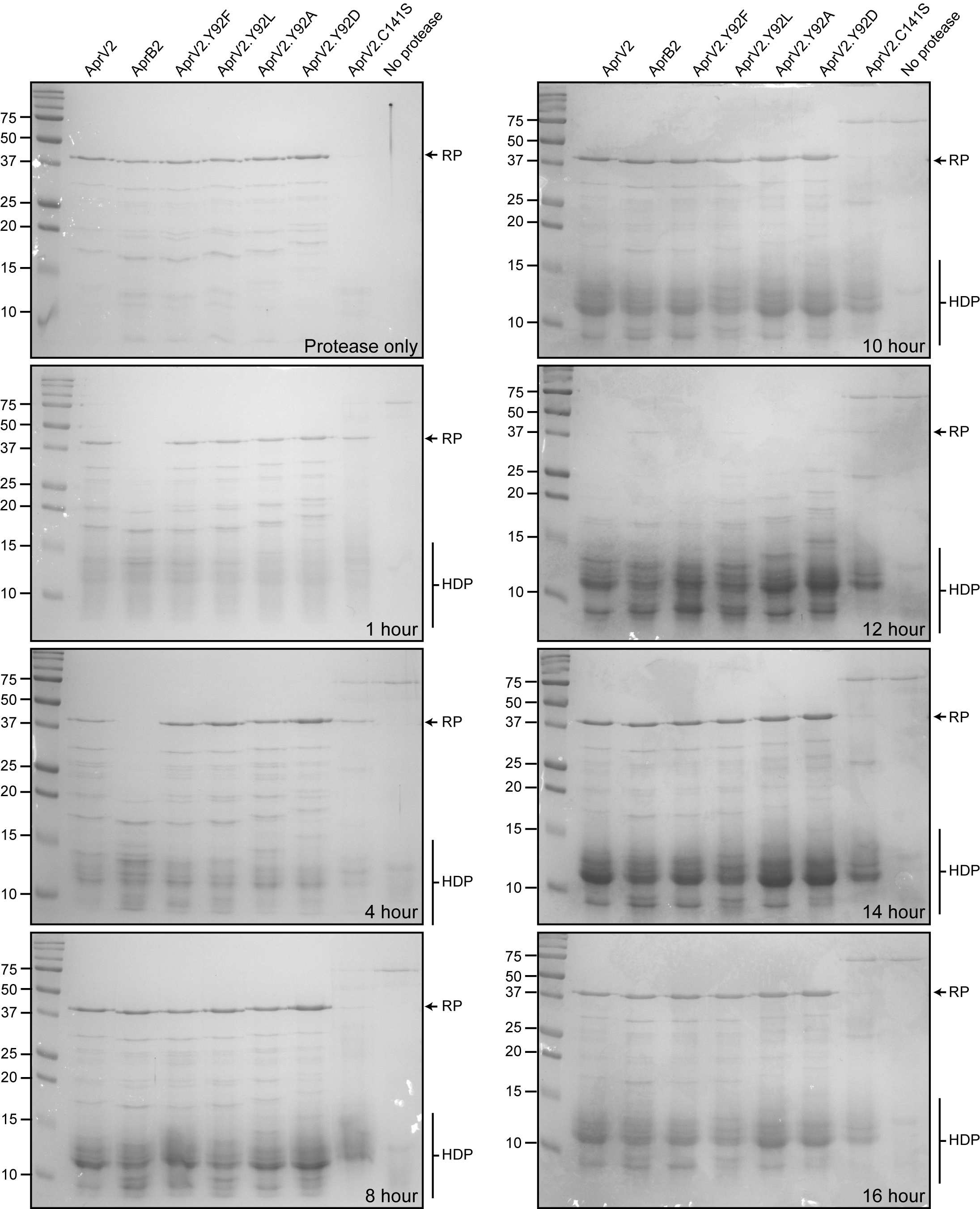

Supplement: Figure S5 — Degradation of sheep hoof by AprV2 and mutants. Purified protease was incubated with hoof material from a disease free sheep at 25°C. The degradation over time was monitored by SDS-PAGE analysis. Degradation products were observed between 5–15 kDa. RP: recombinant protease; HDP: hoof degradation products. (5.95 MB TIF) [file ppat.1001210.s005.tif]

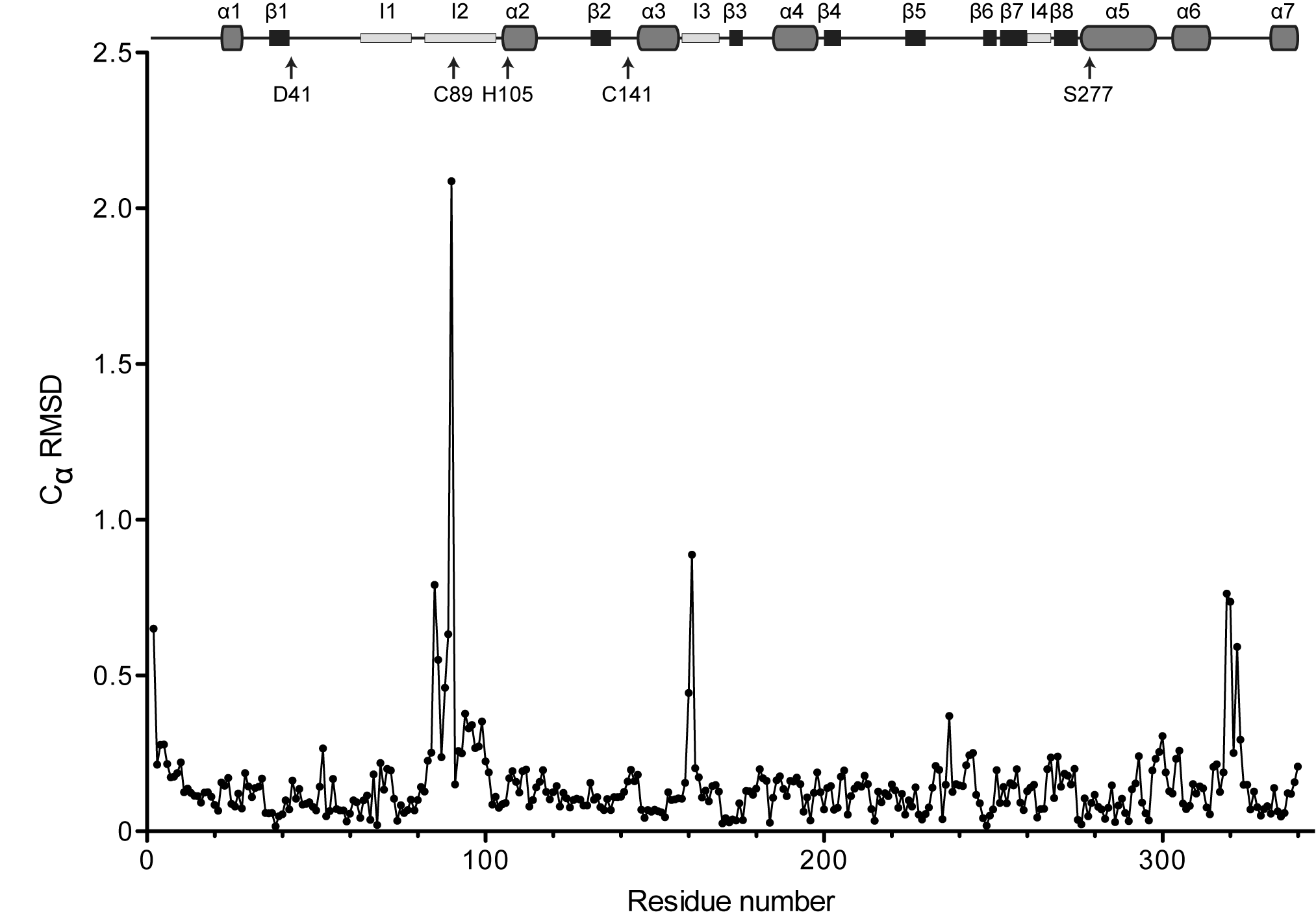

Supplement: Figure S6 — AprV2.C141S is structurally similar to AprV2. The crystal structure of AprV2.C141S and AprV2 were superimposed using LSQKAB in CCP4 [33], [34]. The Cα RMSD for each residue is plotted. The secondary structure of each residue is indicated with α-helices shown in dark grey and β-strands shown in black. The loop insertions are shown in grey and labelled. The location of the catalytic triad (Asp41, His105 and Ser277) and the disrupted disulphide bond (Cys89 and Cys141) is labelled. Secondary structure elements were calculated using stride [53]. (2.89 MB TIF) [file ppat.1001210.s006.tif]
