# Supplementary material for: The Subtilisin-Like Protease AprV2 Is Required for Virulence and Uses a Novel Disulphide-Tethered Exosite to Bind Substrates
Source: PLoS Pathog. 2010 Nov 24;6(11):e1001210. doi: 10.1371/journal.ppat.1001210 (PMC2991261; doi:10.1371/journal.ppat.1001210)
Supplement: Table S1 — Data collection statistics for AprV2.C141S. (0.05 MB DOC) [file ppat.1001210.s007.doc]

***Table S1:*** ***Data collection statistics for AprV2.C141S***

| **Parameter** | **AprV2.C141S** |
| --- | --- |
| Wavelength (Å) | 1.5418 |
| Space group | *P*1 |
| Cell dimensions |  |
| *a*, *b*, *c* (Å) | 42.96, 45.66, 47.25 |
|     α, β, γ  (°) | 97.9, 115.1, 113.4 |
| Resolution (Å) | 30.29 - 2.1 (2.21 - 2.1) * |
| *R*_merge_ | 0.092 (0.56) |
| *I* / σ*I* | 11.4 (2.5) |
| Completeness (%) | 93.6 (91.1) |
| Redundancy | 3.8 (3.8) |

*Values in parentheses are for highest-resolution shell.
